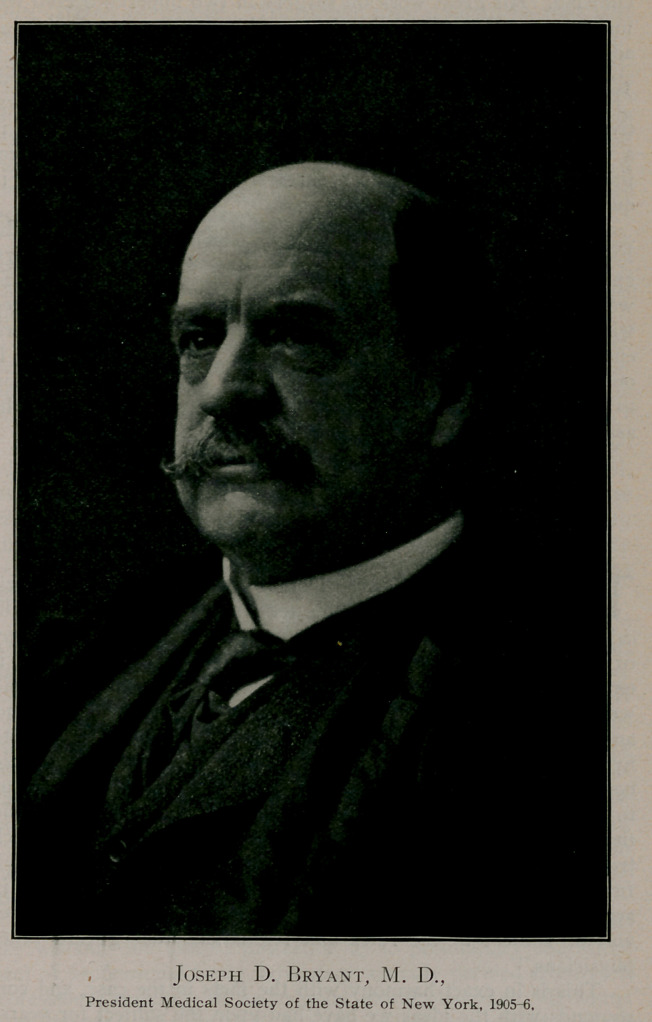# Medical Society of the State of New York

**Published:** 1905-03

**Authors:** 


**A Monthly Review of Medicine and Surgery.**

*EDITOR:*

WILLIAM WARREN POTTER, M. D.

All communications, whether of a literary or business nature, books for review and exchanges, should be addressed to the editor 284 FRANKLIN ST., BUFFALO, N. Y.

VOL. XLIV.—LX. MARCH, 1905. No. 8

### Medical Society of the State of New York

NINETY-NINTH ANNUAL SESSION.

DR. HAMILTON D. WEY, president of the society, has ample reason to feel great satisfaction in the ninety-ninth annual meeting of the Medical Society of the State of New York, held at Albany, January 31, February 1 and 2, 1905. Could the fathers who organised this body in 1806 have looked down upon it while in session during the closing of the first century of its existence, they surely would have been justified in exclaiming, Behold the work which we founded! Verily, we builded better than we knew. So, too, would the distinguished father of the learned president, who occupied the chair in 1872, have been justified in exclaiming, Well done, good and faithful son!

Some special features of the program deserve mention, first of which was the report of the committee of conference. This was presented by the chairman, Dr. Henry L. Elsner, of Syracuse, who gave a summary of the work relating to amalgamation done during the past three years. In the course of the report it appeared that the Medical Society of the State of New York and all its constituent societies had unanimously ratified the propositions of the committee, but that in the Association there was opposition from the county of Onondaga. The legal technicalities were considered and it was stated to be the conclusion of the committee that, to avoid all such in future, it would be best to reratify, and this was done at a special meeting held on Thursday noon after the adjournment of the regular session.

At the conclusion of Chairman Elsner's report, Dr. D. B. Saint John Roosa offered the following resolution, which was passed unanimously: That the committee of the society heretofore appointed for the purpose of bringing about consolidation—namely, Drs. Henry L. Elsner, Abraham Jacobi, Albert Vander Veer, George Ryerson Fowler and Frank Van Fleet, be and they are hereby continued as such committee with full power and authority to do whatever may be necessary to carry the agreement into effect.

Dr. John H. Pryor's report on the work done at Raybrook, and his paper on the Recognition of incipient pulmonary tuberculosis, together with the symposium on Cerebrospinal meningitis participated in by W. T. Councilman, H. L. Elsner, A. Jacobi, Charles G. Stockton, A. E. Davis, Morris Manges, DeLancey Rochester, E. Libman and E. D. Fisher, were among the interesting features of Tuesday.

In the evening Dr. Charles Harrington, of Boston, delivered an address dealing with the origin and development of the various Massachusetts state laboratories and giving in detail what they are accomplishing for the public health. Following this, Dr. Hamilton D. Wey delivered the president's anniversary address, which is published elsewhere in this issue of the JOURNAL. These ceremonies took place in the senate chamber.

The principal feature of Wednesday was a symposium on Prostatism and its treatment, participated in by L. Bolton Bangs, George Ryerson Fowler, Willy Meyer, Charles H. Chetwood, Howard Lilienthal, E. Wood Ruggles, Albert Vander Veer, Samuel Alexander, Willis G. Macdonald, Parker Syms, and (by invitation) Paul Thorndike and Francis H. Watson, of Boston, and Hugh H. Young, of Baltimore. This constituted the most extensive presentation of this topic yet made in a general medical society in this country.

The banquet on Wednesday evening was as usual very largely attended and the speech of Dr. Roosa may be regarded as the special feature of that occasion. The dinner to the ex-presidents, given by Dr. Willis G. Macdonald, at the Fort Orange Club, Monday evening; the dinner by Dr. Vander Veer to the state medical examining board, and the reception by Dr. Neuman on Tuesday evening were among the other social functions of interest. Dr. Neuman's reception was planned as a compliment to Dr. L. S. McMurtry, president of the American Medical Association. Unfortunately, however, Dr. McMurtry was prevented from attendance upon the meeting and participating in its social and scientific functions on account of illness. Telegrams and messages of sympathy, expressing regret at his absence, were duly dispatched to his home at Louisville.

The following-named officers were elected to serve for the ensuing year: president, Joseph D. Bryant, New York; vice-president, Herman R. Ainsworth, Addison; secretary, Frederic C. Curtis, Albany; treasurer, O. D. Ball, Albany.

JOSEPH D. BRYANT, M. D.,

President Medical Society of the State of New York, 1905-6.

President-elect, Joseph D. Bryant, is too well known to the medical profession of the world to need formal introduction to any medical congress or assembly. He is one of our best-known surgeons, being famous as a teacher and author, as well as a clinician. His great work on surgery in two imperial octavo volumes is to be found in the library of every surgeon, and is an accepted textbook in most American medical schools. When it appeared in a new edition a few years ago the JOURNAL said: "Bryant is a forceful writer and makes himself clear in all operative technic. . . . He tells how to control hemorrhage in a way that cannot mislead even the novitiate. . . . His treatise is a distinct addition to a surgical literature, already rich in quantity and quality."

Dr. Bryant ranks as one of the distinguished citizens of the Empire state, prominent in its civic, medical, and social affairs, and his administration of the business interests and scientific work of the society during the current year may be anticipated with a confidence in its successful accomplishment. His experience is large, his acquaintance numerous, and his views broad. It ought to be possible, during his term of office, for Dr. Bryant to unite the medical profession, represented in the society and the association, into a compact harmonious body.

## Figures and Tables

**Figure f1:**